# The Big Potential of Small Particles: Lipid-Based Nanoparticles and Exosomes in Vaccination

**DOI:** 10.3390/vaccines10071119

**Published:** 2022-07-13

**Authors:** Marina Ben Shimon, Shiran Shapira, Jonathan Seni, Nadir Arber

**Affiliations:** 1 The Health Promotion Center and Integrated Cancer Prevention Center, Tel Aviv 6423906, Israel; Marina@covend24.com (M.B.S.); shiranshapira@gmail.com (S.S.); jonathanseni@mail.tau.ac.il (J.S.); 2 Department of Molecular Genetics and Biochemistry, Sackler Faculty of Medicine, Tel Aviv University, Tel Aviv 6423906, Israel

**Keywords:** exosomes, nanotechnology, adjuvant, vaccines

## Abstract

Some of the most significant medical achievements in recent history are the development of distinct and effective vaccines, and the improvement of the efficacy of previously existing ones, which have contributed to the eradication of many dangerous and life-threatening diseases. Immunization depends on the generation of a physiological memory response and protection against infection. It is therefore crucial that antigens are delivered in an efficient manner, to elicit a robust immune response. The recent approval of COVID-19 vaccines containing lipid nanoparticles encapsulating mRNA demonstrates the broad potential of lipid-based delivery systems. In light of this, the present review article summarizes currently synthesized lipid-based nanoparticles such as liposomes, lipid-nano particles, or cell-derived exosomes.

## 1. Introduction

The emergence of diseases such as severe acute respiratory syndrome (SARS), Middle East respiratory syndrome (MERS), and coronavirus disease 2019 (COVID-19), and the subsistence of diseases known for a long time, such as Ebola, Zika, HIV, tuberculosis, and all types of cancers, have driven the development of a massive vaccination industry over the last four decades. The global vaccine market is projected to generate $125.49 billion by 2028 at a CAGR of 10.8% in the forecast period, 2021–2028 [1].

Immunization continues to be the most successful and cost-effective approach to eradicate many diseases. By definition, the basic conditions of a potential vaccine should be to induce a sufficient immunogenic response, yielding a protective umbrella for the host, with minimal adverse effects.

The development of subunit vaccines (*second generation*) has brought great advances, due to marked improvement in safety and physical tolerance in comparison to the traditional attenuated or killed whole-organism approaches (*first generation*). However, subunit vaccines generate a weak immune response due to the use of only a specific part of the pathogen structure. By contrast, RNA or DNA vaccines (*third generation*) induce in situ expression of antigens after immunization, priming immune responses against specific pathogens [2,3,4,5]. Examples of these technologies include mRNA-based vaccines which were developed by BioNTech/Pfizer (*BNT162b2*) [6], and by Moderna (mRNA-1273 vaccine) [7], to address the challenges created by the COVID-19 pandemic, followed by adenovirus-based vaccines from Astra Zeneca [8], Johnson & Johnson (Ad26.COV2.S), and Gamaleya (Sputnik V; 10).

The authorization of mRNA-based vaccines during the pandemic has delivered a platform for the development of vaccine therapies in a relatively simple and affordable manner. One critical factor for a successful pandemic-level vaccine, beyond its biological efficacy, is the manufacturing cost, because billions of vaccines are imperative in a very short time.

Despite the great efforts to achieve more effective vaccine platforms, there remain several unmet medical needs, particularly against challenging pathogens such as *Mycobacterium tuberculosis* and the human immunodeficiency virus (HIV), for which only vaccines with limited efficacy have been produced thus far [9]. The *third generation* vaccines have distinguishable potential in addressing these unmet conditions; however, the biggest challenge preventing the widespread clinical application is an efficient delivery system for mRNA molecules [10].

Lipid nanoparticles (LNPs) were utilized successfully to deliver the nucleic acid cargos in the COVID-19 vaccines. Herein, we review the up-to-date LNP-based technologies and the exciting emerging platform for the extracellular vesicles.

## 2. Ex Vivo-Prepared Lipid-Based Nanoparticles

### 2.1. Liposomes

**Figure 1:**
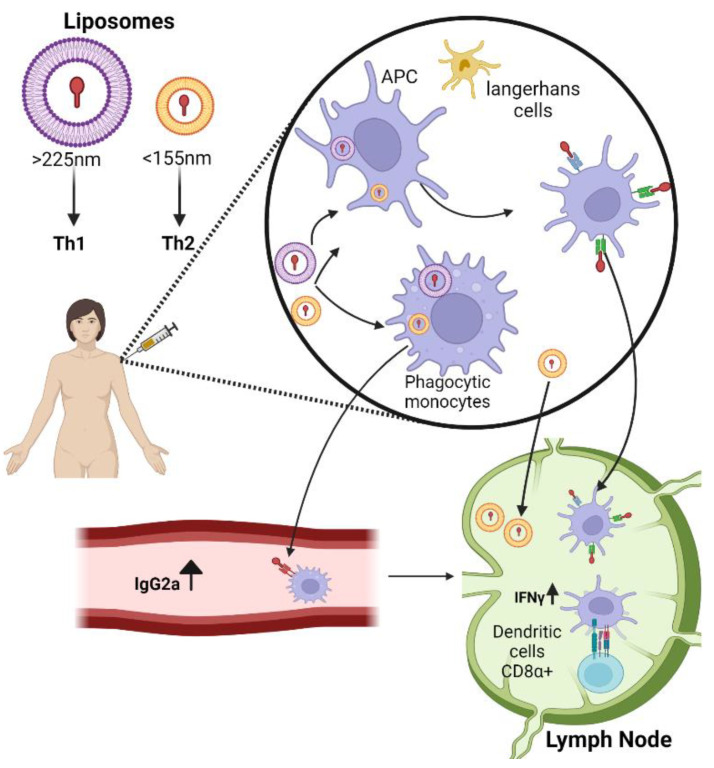
Nanoparticle trafficking from skin to draining lymph node in size-dependent manner. Large particles shuttle from the interstitial space through DC take up, involving activation of cell adhesion molecules, and inducing preferentially Th1 response (elevation of IgG2a in the plasma) and elevation of IFNγ in the lymph node. Small nanoparticles drain freely to the lymphoid node and induce Th2 response of increased IgG1 and IL5. Created with BioRender.com.

Liposomes are the earliest nanoparticle delivery platform. They consist of one (unilamellar) or more (multilamellar) phospholipid bilayers surrounding an aqueous core that house the drug of interest and resemble biological membrane [11]. Liposome composition and preparation can be tailored according to the desired features such as lipid composition, charge, size, entrapment, and location of antigens or adjuvants [12]. The intrinsic adjuvanticities of liposomes have long been confirmed and, unlike other adjuvants, they have shown minimal reactogenicity and few cases causing hypersensitivity-associated reactions in immunized subjects [13]. This can be attributed to their size and shape, which mimics pathogenic microbes and some subcellular structures, leading to the arousal of strong eliminatory mechanisms via both humoral and cellular responses [14]. Indeed, the size of many common viruses that are freely able to drain the lymph node is between 30–200 nm [15]. The liposome dimensions can impact their adjuvant efficacy, and several studies have shown that either Th1 and Th2 responses can be evoked by variations in particle size. Specifically, in cases of big vesicle vaccination (>225 nm) a significantly higher Th1 response has been reported, whereas the same antigen encapsulated in small liposomes (<155 nm) induced a prevalent Th2 response [16]. This size-dependent immune effect is attributed primarily to their individual modes of entry into lymph nodes. Smaller particles freely penetrate the draining lymph node whereas larger vesicles are internalized by tissue-dependent resident dendritic cells. Vania Manolova et al. demonstrated that, for the association of large particles with monocyte-derived DC, there must be cell-associated transport. In contrast, LN-resident CD8α^+^ DCs were mostly associated with a small particle (Figure 1). Larger vesicles delayed clearance, resulting in prolonged exposure time of antigens at the injection site (depot effect) [17]. As mentioned above, liposomal charge must also be considered. Cationic liposomes are preferably utilized as vaccine carriers, since the positive charge provides reduced clearance rate, prolonged exposure time of antigen at the mucosal surface (depot effect), and enhanced endocytosis of liposomes by APC [18]. In addition, positively charged liposomes demonstrate enhanced adjuvanticity over neutral and negatively charged liposomes [19]. Several liposome adjuvants have been licensed for human use and others are being evaluated in clinical trials. In 1995, the FDA approved the first nano-drug (Doxil), a doxorubicin-loaded liposome utilized in the treatment of cancers [20]. Commercially available vaccines include Cervex^®^, Inflexal^®^, and Epaxal^®^, against infection by human papilloma virus, influenza virus, and hepatitis A infection, respectively [21]. Liposomes paved the way for the application of nanotechnology as drug and vaccine carriers, and the subsequent development of improved derivatives such as lipid nanoparticles.

### 2.2. Lipid Nanoparticles

**Figure 2:**
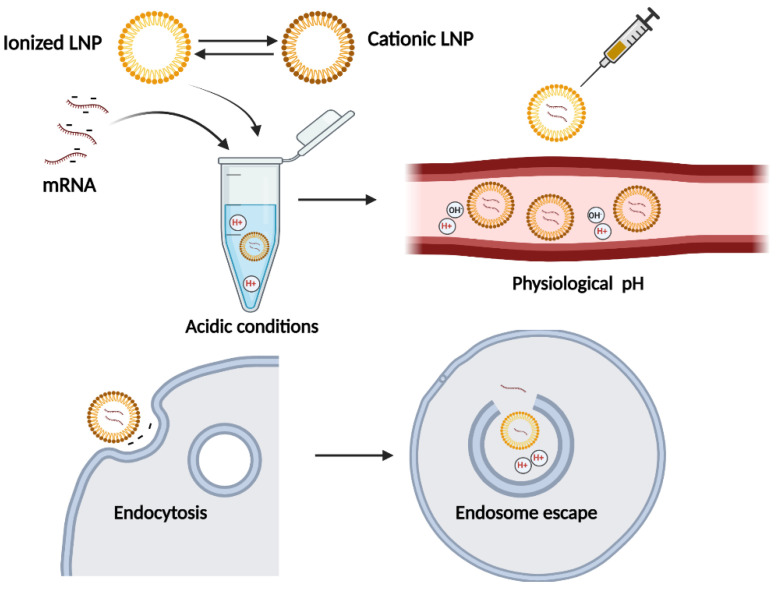
Lipid nanoparticles’ ionizable lipid component facilitates the delivery of mRNA cargo. The transition of LNPs between positive and neutral charges from the mRNA loading step to the final release to the cell cytoplasm is shown. In acidic condition, ionizable lipids are positively charged, which promotes mRNA loading. Then, in the systemic circulation, they became neutral positive, which lowers their toxicity and prevents rapid sequestration by immune cells. The slightly positive charge facilitates particles’ entrance to the cells by endocytosis. Upon acidification in the endosome, the particles became positive again, which induces hexagonal phase structures, disrupting the membrane of the endosome. Created with BioRender.com.

Both liposomes and LNPs are utilized as drug delivery vehicles in the body; however, LNPs only have a single phospholipid outer layer encapsulating the interior. LNPs usually have four components: 1—ionizable or cation lipid, which allows endosomal release of mRNA to the cytoplasm; 2—lipid-linked polyethylene glycol (PEG) which increases the half-time of formulations; 3—cholesterol as stabilizing agent; 4—naturally occurring phospholipids which support bilayer structure. This provides improved stability of the cargo, a rigid morphology, and more efficient cellular penetration [22,23]. Two mRNA-based COVID-19 vaccines were developed using these lipid nanoparticles. The high biological safety profile of mRNA-based vaccines or therapies is a prominent advantage, since the biggest concern with nucleic acid therapeutics is the risk of permanent change in the genome. mRNA is noninfectious, non-integrating, and its in vivo half-life can be regulated using various modifications and delivery methods. mRNA is degraded by normal cellular processes manifested by abundantly available enzymatic machinery, and naked RNA is rapidly degraded by extracellular RNases [24]. However, its high degradability is also the biggest challenge in utilizing mRNA molecules in therapeutics, because its sufficient expression is dependent on stable and efficient distribution. With this aim, lipid-based nanoparticles were adopted with improved formulation. The key to success of the NLPs utilized in COVID-19 vaccines was the ionized lipid substance that switches charges according to the environmental pH. The NLP is positively charged during production to improve the mRNA complexation in acidic buffer, but it converts to neutral charge under physiological conditions that reduce toxicity post-infection. Since biological membranes and nucleic acids are negatively charged, it is difficult to deliver mRNA across this barrier; the switch to the near-neutrally charged NLP at physiological pH facilitates the mRNA cell penetration. Subsequently, the NLP switches again to positive as the pH in the endosome drops, which is crucial for endosome escape for effective intracellular delivery (Figure 2) [24,25,26,27,28]. Upon intramuscular injection of mRNA loaded LNPs vaccines, particles can be either internalized by interstitial cells or drained directly to the lymph node. There are few optional cell types for mRNA translation, including somatic cells, resident or recruited APCs (antigen-presenting cells) in the interstitial space, or in the lymph node, by various immune cells reside, including naïve T and B cells. Subsequently, the expressed spike antigen can either be degraded and presented on MHC-1, which then binds the epitope to CD8^+^ T cells, or endocytosed by APCs. APCs present the epitope by MHC II for CD4^+^ cells. In addition, secreted spike antigens can be internalized by B-cell receptors [29]. Although the optimized formulations of the ionizable lipid replacing the permanent cationic lipid were expected to be less toxic, there was still evidence of side effects indicative of acute inflammation. Previously published research illustrated that empty LNPs caused an innate immune response, despite the presumption that this vaccine platform was primarily noninflammatory. The inflammation consisted of leucocyte infiltration, activation of inflammatory pathways, and cytokine secretion. Thus, LNPs can serve as particle-carriers with adjuvant activity [30]. However, the balance of positive and negative inflammatory properties should be evaluated, since there is a possibility of exacerbating potential side effects due to the robust inflammatory milieu induced by LNP combined with presentation of vaccine-derived peptides outside of APC.

## 3. Cell-Derived Nanoparticles: Exosomes

Among the variety of delivery systems created with the aim of increasing antigen presentation and enhancing immune response, cell-derived exosomes have emerged as a novel platform for vaccine delivery [31,32]. Exosomes are naturally occurring vesicles. Exosomes are nanoparticles that can range in size from 30–200 nm, and are produced by almost all cell types. Exosome biogenesis begins with the cell membrane budding inward, followed by endosome invagination, which results in the formation of multi-vesicular bodies that are then secreted into the extracellular space as exosomes [33,34]. Exosomes can be designed to exhibit specific ligands on their surface to target particular cells, and can be loaded with diverse drugs which are located either on the membrane surface or carried within the exosome for protection from degradation. They exhibit low immunogenicity, significantly less toxicity than lipid nanoparticles, improved drug encapsulation coupled with a controlled release, and greater in vivo biodistribution [32,35]. Recent reports established critical roles for exosomes in both physiological and pathophysiological processes, including host–pathogen interaction [36], cell–cell communication [37], genetic exchange between cells [38], and infectious agent transport [37,39].

An evolving field of “Exo-vaccination” relies on dendritic cell-derived exosomes that consist of proteins involved in the immune response. The idea originated from dendritic cell-based immunotherapy, which has manufacturing limitations on mass production, definition of quality controls parameters, and long-term storage [40,41]. Dendritic cell (DC)-derived exosomes are an attractive substitute for whole DC culture. In addition, GMP laboratory procedures for exosome harvesting and purification have been set up for clinical implementations [42,43]. Exosomes secreted from professional antigen-presenting cells (B lymphocytes and dendritic cells) are enriched with immunomodulatory proteins such as: MHC Class I and II complexes, costimulatory molecules, HSP70–90, and chaperons [40,44,45]. Two strategies are available for MHC peptide presentation on DC-derived exosomes, naturally occurring following cell culture activation or direct loading of peptide, with the latter method being deemed more efficient [46]. Preclinical studies have been conducted in two phase I studies on cancer patients immunized with DC-derived exosomes presenting tumor-derived peptides. Phase I clinical trials were conducted with DC-based vaccination in melanoma patients. Exosomes, purified from DC cultures obtained from patients’ leukapheresis, were loaded efficiently in an acidic environment with MHC Class I or II peptides. The exosomes were safe, and did not cause any related side effects. The observed immune response following exosome treatment manifested enhanced NK cell effector functions [47]. Exosomes can be loaded with mRNA molecules to express the immunogenic antigen of interest. In contrast to LNP, which elicited cellular toxicity, exosomes have no adverse effect. Shang Jui et al. demonstrated production of 293Hek cell line-derived exosomes loaded with mRNA-expressing immunogenic antigen. With in vitro and in vivo models of mRNA exosome loading, the mRNA antigen was expressed and induced both humoral and cellular responses [48,49].

Despite the progress in the field, the need to improve efficient exosome cargo uptake, to optimize tropism and biodistribution, and to inhibit lysosomal destruction activity, continues to be a challenge in exosome therapy.

There are currently no FDA-approved exosome products for human use in the USA. According to the FDA, exosomes are classified as a product that requires studies regarding safety and efficacy, the purity of the product, and its power in treating a specific medical condition. Therapies using exosomes are under the Investigational New Drug (IND) developmental phase, and require the approval of the regulatory agencies before initiating the clinical trial [50]. The absence of standard regulatory guidelines for manufacturing exosome-based drugs is a significant obstacle that must be overcome. In the cases of protein-, cell-, molecules-, and nanomaterials-based therapies, the requirements for product characterization are abundantly available. However, exosomes don’t belong to any of these categories, halting the progress of such therapies to advanced stages in clinical trials.

Nevertheless, several exosome-based drug formulations are currently in clinical trials [51]. Up until April 2022, we have found 258 clinical trials in which exosome-based formulations are applied [52]. Out of the 258 trials, 111 involve cancer-related studies, 21 are associated with brain pathologies, and 120 include diabetic, cardiovascular, lung, and kidney diseases. In addition, 16 trials are for COVID-19 clinical studies. Table 1 demonstrates the trends of exosome-based therapies in clinical trials.

### 3.1. Exosome-Based Therapies for COVID-19 in Clinical Trials

At the present time, COVID-19 has been spreading across the world, and outbreaks continue to occur. It is imperative to find a safe and effective therapeutic approach for COVID-19 patients, and exosomes bring attractive possibilities as diagnostic biomarkers, in addition to targeted drug delivery. COVID-19-related clinical trials based on the exosome platform confirm its flexible application and capability. This section will discuss several examples.

To explore the safety and efficiency of aerosol inhalation of exosomes derived from allogenic adipose mesenchymal stem cells (MSCs-Exo), single-arm, open-label, combined interventional clinical trials were designed for the treatment of patients hospitalized with novel coronavirus severe pneumonia (NCP) [54]. Blazquez et al. [55] reported that human adipose MSC-derived exosomes (exo-hASCs) induced an inhibitory effect on the differentiation, activation, and proliferation of T cells. In addition, IFN-γ release downregulation on in vitro stimulated cells with anti-CD2/anti-CD3/anti-CD28, showing that exo-hASCs can be considered as therapeutic agents for the treatment of inflammation-related diseases [56].

In a second trial, to test the safety and efficiency of T-cell-derived exosomes by metered-dose inhaler, a single-arm, open-label, combined interventional (phase I/II) clinical trial was designed for the treatment of patients at early stages of novel coronavirus pneumonia [57]. COVID-19-specific T-cells (CSTC) are T cells activated and expanded in vitro by exposing them to viral peptide fragments in the presence of cytokines. These fragmented COVID-19 peptides activate specific T cells, and stimulate the secretion of potent mediators, including IFN-γ in forms of exosomes [58]. It is proposed that the treatment of COVID-19 patients with CSTC-exosomes, at early stages of pulmonary disease, will control disease progression [59,60].

In a third example, a phase I/II randomized, double-blinded, placebo-controlled trial evaluated the safety and potential efficacy of an intravenous infusion of Zofin (Organicell flow) for treatment of moderate to severe acute respiratory syndrome (ARDS) related to COVID-19 infection [61]. Zofin is a cellular product derived from human amniotic fluid. It consists of over 300 growth factors, cytokines, chemokines, and extracellular vesicles/nanoparticles derived from amniotic and epithelial cells. The presence of exosome-associated proteins CD63, CD81, CD9, and CD133 were revealed by surface marker analysis, and the completed sequencing showed 102 commonly expressed miRNA sequences. Proinflammatory cytokines found to be targeted by miRNA include TNF, IL-6, IL-8, FGF2, IFNB1, IGF1, IL36a, IL37, TGF-B2, VEGFA, CCL8, and CXCL12. It has been suggested that inhibition or suppression of this pro-inflammatory cytokine cascade (cytokine storm) may reduce the severity of symptoms associated with elevated immune response [62,63].

In another trial, a nonrandomized open-label cohort study addresses the safety and efficacy of exosomes derived from allogeneic bone marrow mesenchymal stem cells (ExoFlo^TM^; bmMSC-derived exosomes) as intravenous treatment for severe COVID-19 and for moderate-to-severe ARDS [64]. No adverse effects were observed within 72 h of ExoFlo^TM^ administration. Due to its ability to restore oxygenation, to downregulate cytokine storm, and to reconstitute immunity, ExoFlo^TM^ is considered a promising therapeutic candidate for severe COVID-19.

The COVID-19 pandemic outbreak accelerated the development of clinical trials that launched these new therapeutics platforms. This pharmaceutical blooming boosted recognition of exosomal-based therapies, which led to their immense prominence in clinical trials, and subsequently necessitated the creation of regulatory authorities to consolidate guidelines for exosome-based drugs.

### 3.2. Exosome CD24 (EXO-CD24) Delivery System for COVID-19

CD24 is a small, heavily glycosylated mucin-like cell surface protein anchored to the membrane via glycosyl phosphatidylinositol, known to be a natural endogenous negative regulator of the immune system [65]. CD24 associates with DAMPs but not with PAMPs, meaning that it does not interfere with viral clearance. The binding of CD24 to DAMPs prevents them from binding to TLRs; therefore, CD24 inhibits DAMP-activation of the NFκB pathway, a key signaling pathway driving production of cytokines and chemokines [56]. Another distinct class of pattern recognition receptors are Siglecs, which regulate immune cell functions. CD24 binds Siglec-10, resulting in an activation of the Siglec-10 signaling pathway [66,67]. This pathway negatively regulates the activity of NFκB, through the immunoreceptor tyrosine-based inhibition motif (ITIM) domains associated with SHP-1 (SRC homology 2 domain-containing protein tyrosine phosphatase-1). This synergistic effect yields tight inhibition of the NF-κB pathway, thus reducing the likelihood of developing a potentially deadly cytokine storm and leading to a return to immune homeostasis.

We developed a therapeutic drug platform named EXO-CD24, carried by exosomes, as a highly body-compatible delivery vehicle. Exosomes are engineered to overexpress CD24 [68], an endogenous immunomodulator of the immune system, aiming to target the cytokine storm in the lungs of COVID-19 patients.

Mortality in COVID-19 patients has been linked to the presence of the cytokine storm induced by SARS-CoV-2. In about 5% of COVID-19 patients, after a window of 5–10 days, a rapid clinical deterioration may occur that can lead to acute respiratory distress syndrome (ARDS), a life-threatening form of respiratory failure. ARDS is a critical medical condition with an unmet need for therapy for approximately 1.5–79 patients per 100,000 each year in Europe alone, resulting in nearly 25% mortality. In the USA, extrapolation of the data suggests that there are approximately 190,000 cases of ARDS each year. Globally, ARDS accounts for 10% of intensive care unit admissions, representing more than three million patients with ARDS annually. Although the exact mechanism of SARS-CoV-2 in ARDS is not yet fully understood, the induction of cytokine storm is considered to be one of the leading factors. EXO-CD24 may potentially be used as a novel treatment to suppress the hyper-inflammatory response in the lungs of severely affected COVID-19-associated ARDS patients, as well as in other systemic diseases where cytokine storm is developed.

EXO-CD24 is delivered by inhalation, a clinically simple mode of administration that can be administered by non-medical staff, reducing costs during treatment. Inhalation enables a strong reduction of the required dose (as opposed to systemic administration) and reduces the risk for adverse events. In this regard, patients with moderate- to high-severity COVID-19 were recruited in a phase Ib/IIa open-label study conducted in Israel. Participants were given increasing doses (from 1 × 10^8^ to 1 × 10^10^ exosomes per dose) of EXO-CD24 particles for five consecutive days [69]. A fast and significant reduction in the inflammatory markers and in cytokine/chemokine levels confirmed the expected efficacy of EXO-CD24 in downregulating the cytokine storm. No adverse effects related to the drug were observed, indicating an excellent safety profile [70].

Other groups have applied soluble CD24 (CD24Fc) to evaluate hospitalized adult patients with confirmed SARS-CoV-2 infection. They were randomly assigned to receive a single intravenous infusion of CD24Fc 480 mg or placebo [71]. CD24Fc was generally well tolerated, and promoted clinical improvement in hospitalized patients with COVID-19 who were receiving oxygen support. Results suggest that targeting inflammation provides a therapeutic alternative for patients hospitalized with COVID-19 [72,73].

### 3.3. Exosome-Based Therapies—Translational Challenges

However, exosome biogenesis and complex functioning is not yet fully understood, particularly the mechanisms involved in the uptake into the exosomes of the drugs to be transported, and in their release into cells after exosome internalization. An important consideration in applying exosome-based therapy to current clinical practice is the standardization of isolation and storage techniques. At the present time, many exosome isolation kits are on the market, in addition to laboratory-made cocktails and protocols. However, there is no standardization for reagents and for storage conditions for exosome-based preparations. This leads to broad variations in the reproducibility of the results, which generates difficulties in drawing adequate conclusions, making the transition to the clinic problematic [74]. In addition, keeping in mind the necessity for large-scale prospective production, easier and faster methods for exosome separation and purification are needed, along with the development of engineered exosomes to overcome drug-loading issues, and to obtain uniform and stable results in drug delivery applications, both in preclinical and clinical studies. In this regard, pharmacokinetic and pharmacodynamic properties through large-scale prospective research studies will be also required [31].

Despite the significant progress in the field over the last decades, there are questions that need to be addressed. Using scanning electron microscopy (SEM), exosomes can be distinguished from other contaminating extracellular vesicles, based on the size distribution [75]. However, there are still no standard methods to follow and characterize exosomes for in vitro and in vivo studies. In a first approach, Wu et al. developed a new flow cytometry assay to characterize membrane protein expression on exosomes, by using a lipophilic fluorescent tracer dye (DiI; dialkylcarbocyanine dye) to detect low copy-number proteins through unbiased clustering of exosomes. Applying this approach, exosomes derived from SKBR3 cells, a cell model for human HER2+ breast cancer, were shown to contain both HER1 and HER2 proteins, but at very different levels of abundance. The relative densities of HER1 and HER2 on the new assay establishes a consistent framework to characterize exosomes through the identification of specific low-expressing proteins in exosome membrane [75].

Furthermore, there are no universal exosome markers to allow the identification of these vesicles [31,51]. However, a new study reported that exosomes contain a core proteome of approximately 1200 proteins common to exosomes from all cells. Among them, syntenin-1 has been shown to be the most abundant protein across all exosomes, defining it as a potential universal marker for exosomes [76].

## 4. Author Opinion

Exosomes play an innate role in the body by working as a vehicle for the transfer of biological agents between cells, which have the potential to be developed as a shuttle for delivering drugs of therapeutic need, by using their naturally engineered defense mechanisms.

In addition to their utility in infectious diseases, the potential of exosome-based therapy is vast and stretches across many fields of medicine. It has been described for many other conditions, including neurodegenerative disorders [77], autoimmune diseases [78], cardiovascular diseases [79], bone and orthopedic conditions [80], and for cancer diagnosis [81]. It is expected that exosomes will be pivotal in understanding treatment for the unresolved aspects of multiple conditions for which adequate treatment or diagnosis is not yet currently available.

## 5. Conclusions

The application of nanotechnology in immunization constitutes the basis of the healthcare system. The massive growth in this field has allowed the creation of new approaches that are safer and more reliable. Nanotechnology is able to compete with the latest medical treatments by creating new vaccines, adjuvants, and vaccine delivery platforms.

Undoubtedly, there is a necessity to further explore and reevaluate how to make currently available vaccines more effective in creating a robust and long-term immune response for patients, while maintaining a strong safety profile. For this reason, future studies should take exosomes into consideration as one of the emerging platforms for targeted vaccine delivery.

The wide range of biological compounds found and released from exosomes under physiological conditions has useful applications in the context of healthcare and drug delivery. These include the discovery of new biomarkers, to establish new imaging tools, and the development of therapeutic carriers for a broad range of diseases.

## Figures and Tables

**Table 1:** Current submitted clinical trials.

**Exosome Source**	**Disease**	**Loaded Component**	**Rout of Administration**	**Phase**	**End**	**Clinical Trial Identification Number**
MSCs	Coronavirus pneumonia	None	Inhalation	I	2020	NCT04276987
Human placenta MSCs	Complex perianal fistula	None	Fistula tact injection	I/II	Ongoing	NCT05402748
Allogenic MSCs	Acute ischemic stroke	miR-124	Stereotaxis/intraparenchymal	I/II	Ongoing	NCT03384433
MSCs	COVID-19	None	I.V	I/II	Ongoing	NCT04798716
MSCs	COVID-19	None	I.V	II/III	Ongoing	NCT05216562
Mesenchymal progenitor cell	Microbial pulmonary infection	None	Inhalation	I/II	Ongoing	NCT04544215
Mesenchymal stromal cells	Pancreas cancer	KrasG12D siRNA	I.V	I	Ongoing	NCT03608631
MSCs	Epidermolysis bullosa	None	Dermal	I/II	Enrolled	NCT04173650
Adipose MSCs	Alzheimer	None	Nasal drip	I/II	Ongoing	NCT04388982
MSCs	COVID-19	None	Inhalation	I/II	2020	NCT04491240
Autologous adipose-derived stem cells	Periodontitis	None	Periodontal pockets injection	I	Ongoing	NCT04270006
Umbilical cord blood-derived MSCs	Type I diabetes mellitus	None	I.V	I/III	Ongoing	NCT02138331
MSCs	Knee osteoarthritis	None	Intra-articular injection	I	Ongoing	NCT05060107
Autologous plasma	Cutaneous ulcers	None	Dermal	I	Ongoing	NCT02565264
Platelet-rich plasma (PRP) enriched with exosomes	Chronic low back pain	None	Nucleus pulposus	I	Ongoing	NCT04849429
Dendritic cells	Non-small cell lung cancer	Tumour antigen	n.d	II	2018	NCT01159288
T cell	COVID-19	None	Inhalation	I	Ongoing	NCT04389385
Hek293 cell line	COVID-19	CD24 *	Inhalation	II	Ongoing	NCT04969172

Data retrieved from [53]. Abbreviations: MSCs, mesenchymal stem Cells. * Exosomes presenting CD24 protein on their surface.

## Data Availability

Not applicable.
